# Rezidivierende Livedo-artige Hautveränderungen nach einer Vektor-basierten COVID-19-Impfung

**DOI:** 10.1007/s00105-023-05160-3

**Published:** 2023-05-26

**Authors:** Teresa Kränke, Urban Cerpes, Franz Legat, Emad Arbab, Birger Kränke

**Affiliations:** Universitätsklinik für Dermatologie und Venerologie, Medizinische Universität Graz, Graz, Österreich

**Keywords:** Urtikaria, Nebenwirkung, Kutan, Immunisierung, Infekt, Urticaria, Adverse event, Skin diseases, Immunization, Infection

## Abstract

Die COVID-19-Pandemie stellt das weltweite Gesundheitssystem seit 2020 vor einzigartige Herausforderungen. Faszinierend und v. a. von besonderer gesundheitspolitischer Bedeutung ist es, dass es kaum 1 Jahr nach den ersten Berichten über COVID-19-Infektionen mehreren Forschungsgruppen gelungen ist, Impfstoffe gegen das Coronavirus zu entwickeln. Derzeit sind 3 Klassen von Impfstoffen verfügbar (mRNA-Impfstoffe, Vektorimpfstoffe und Impfstoffe, die das gesamte inaktivierte Virus enthalten). Wir berichten über eine Patientin, die kurz nach der ersten Gabe des Coronaimpfstoffes von AstraZeneca/Oxford (ChAdOx1) rötliche, teilweise urtikarielle Hautveränderungen am rechten Arm und der rechten Flanke entwickelte. Die Läsionen waren flüchtig, rezidivierten allerdings in loco und anderen Lokalisationen über einige Tage. Das klinische Bild war ungewöhnlich. Eine Unterscheidung zwischen einer – für uns am ehesten wahrscheinlichen – Impfstoff-getriggerten akuten Urtikaria und einem urtikariellen Exanthem war uns nicht sicher möglich.

## Abstract (en)

Starting in 2020, the global health system faced unprecedent challenges due to the coronavirus disease 2019 (COVID-19) pandemic and the consequences are still felt. All the more fascinating and of particular importance for health policy was the development of potent vaccines within about one year by several research groups after the first reports of COVID-19 infections. To date, three types of COVID-19 vaccines are available, i.e., messenger RNA-based vaccines, adenoviral vector vaccines, and inactivated whole-virus vaccines. We report a woman who developed reddish, partially urticarial skin lesions on her right arm and flank shortly after the first dose with the corona vaccination from AstraZeneca/Oxford (ChAdOx1). The lesions were transient, however reoccurred in loco and at other locations over several days. The clinical presentation was unusual and was correctly assigned due to the clinical course.

## Anamnese

Eine 30-jährige Patientin stellte sich im März 2021 in unserer Notaufnahme vor und berichtete über das plötzliche Auftreten von nicht juckenden rötlichen Hautveränderungen an ihrem rechten Arm und der rechten Flanke. Die Hautveränderungen seien 6 h zuvor erstmals aufgetreten und nähmen seither an Größe zu. Systemische Symptome wie Fieber, Muskel- oder Gelenkschmerzen wurden verneint. Ebenso wenig waren eine regelmäßige bzw. sporadische Einnahme von Medikamenten oder ein rezenter Infekt vor Auftreten der Hautveränderungen zu erheben. Die Patientin berichtete weiterhin, dass sie unter einer Hausstaubmilbenallergie leide und diesbezüglich saisonal (in den Wintermonaten) H_1_-Antihistaminika (Histamin-1 Rezeptorantagonist) einnehme.

In der erweiterten Anamnese ließ sich erheben, dass die Patientin 3 h vor Auftreten der Läsionen ihre erste Coronaimpfung mit dem Impfstoff von AstraZeneca/Oxford (ChAdOx1) in den rechten Oberarm erhalten hatte.

## Befund

**Figure 1:**
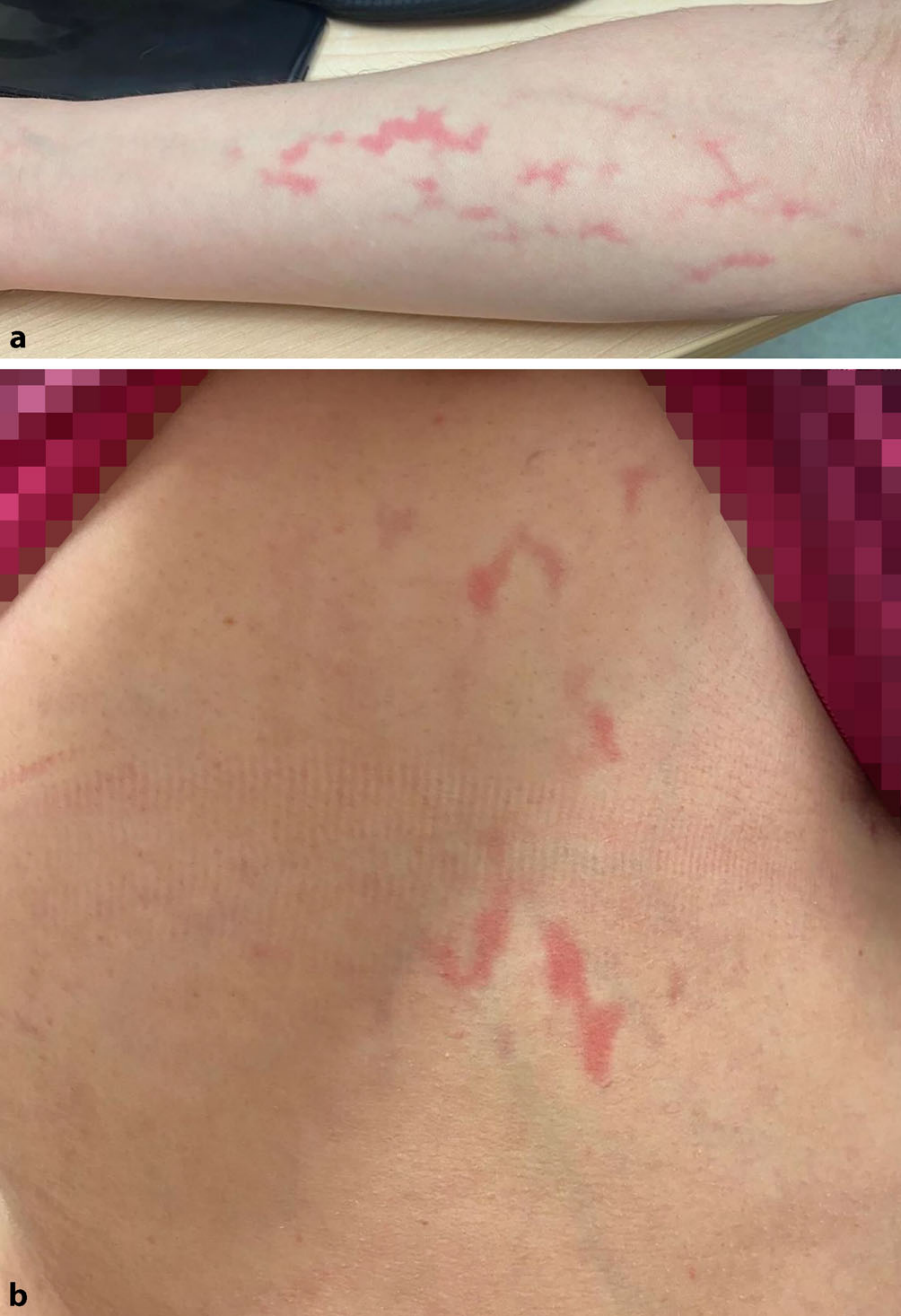

Bei der klinischen Untersuchung zeigten sich rötliche, nicht wegdrückbare, retikuläre, teilweise urtikarielle Läsionen am rechten Arm und der rechten Flanke (Abb. 1a, b). Das übrige Integument einschließlich Kapillitium, Mund- und Genitalschleimhaut sowie den palmoplantaren Flächen war nicht befallen. Die Injektionsstelle der zuvor erhaltenen Impfung war ebenfalls erscheinungsfrei.

## Therapie und Verlauf

Da das klinische Bild initial nicht eindeutig einzuordnen war, bestellten wir die Patientin am Folgetag zu einer klinischen Kontrolle. Die am Vortag aufgetretenen Läsionen waren in der Zwischenzeit abgeblasst, allerdings kamen seither umbilikal ähnliche, ebenfalls nicht juckende Läsionen hinzu. Die Patientin gab weiterhin Wohlbefinden an. In den durchgeführten Laboruntersuchungen zeigten sich eine Leukopenie (2,89 10^9^/l), Lymphopenie (0,4 10^9^/l) sowie ein gering erhöhtes C‑reaktives Protein (7,6 mg/l); das übrige Routinelabor (hier insbesondere die Thrombozytenzahl) inklusive klinischer Chemie war unauffällig.

In den folgenden 10 Tagen kam es wiederholt zu gleichartigen, retikulären Hautveränderungen am rechten Arm, umbilikal, inframammär und abdominal; die Läsionen bestanden jeweils für maximal 24 h und verschwanden nach dieser Zeit spontan ohne jegliches Rezidiv bis aktuell. Nach Rücksprache mit der hämatologischen Abteilung wurden obige Laborveränderungen kontrolliert und normalisierten sich innerhalb 1 Woche. Zur weiteren Abklärung sowie zum Ausschluss einer Gerinnungsstörung führten wir eine Reihe von weiteren Laboruntersuchungen durch (Thrombophiliescreening inklusive Faktor-V-Leiden-Mutation und Antiphospholipidantikörper), die sich unauffällig zeigten.

Um zudem eine mögliche Sensibilisierung auf das potenzielle Allergen Polysorbat 80 des Impfstoffes von AstraZeneca/Oxford auszuschließen, wurden bei der Patientin in vivo Allergietestungen mit Polysorbat 80 (Pricktest 1:1; Intrakutantest 1:100, 1:10 und 1:1) durchgeführt, die negative Ergebnisse zeigten.

## Diagnose

Aufgrund des klinischen Befundes und besonders des Verlaufs gehen wir davon aus, dass unsere Patientin an einem retikulären urtikariellen Exanthem litt, das sich klinisch Livedo-artig präsentierte.

## Diskussion

Die COVID-19-Pandemie („coronavirus disease 2019“) stellt eine beispiellose Herausforderung für die individuelle Gesundheit und die weltweiten Gesundheitssysteme dar, sodass potente Impfstoffe benötigt werden, um diese Krise zu beenden. Derzeit sind 3 verschiedene Gruppen von COVID-19-Impfstoffen verfügbar, nämlich messenger-RNA-basierte Impfstoffe, Vektorimpfstoffe sowie Impfstoffe, die das gesamte inaktivierte Virus enthalten [1]. Diese Impfstoffe sind bis heute milliardenfach verabreicht worden, sodass konsequenterweise auch die Zahl an Berichten über unerwünschte (Neben‑)Wirkungen zugenommen hat. Diese reichen von mild und üblicherweise selbstlimitierend (etwa Lokalreaktion an der Injektionsstelle) bis zu schwerwiegend und potenziell lebensbedrohlich (etwa anaphylaktoide Reaktionen oder die Vakzin-induzierte immunthrombotische Thrombozytopenie) [1–3].

Eine aktuelle Studie [3] hat sich mit der großen Variabilität an kutanen unerwünschten Nebenwirkungen nach einer COVID-19-Impfung bei mehr als 400 Patienten befasst. Die Autoren konnten zeigen, dass Lokalreaktionen an der Injektionsstelle, Urtikaria und morbilliforme Exantheme die häufigsten kutanen Nebenwirkungen in allen Impfgruppen (messenger-RNA- und Vektor-basiert) waren. In der Gruppe der Patienten, die mit dem Vektor-basierten Impfstoff von AstraZeneca geimpft wurden, war eine Urtikaria mit 21,1 % die am häufigsten beobachtete Nebenwirkung.

Das Exanthem bei unserer Patientin war sehr wahrscheinlich getriggert durch die (zu erwartende) Vakzin-induzierte Immunreaktion und ist möglicherweise auch im Kontext mit einer subklinischen (nicht-COVID) Virusinfektion zu betrachten. Ob es sich bei dem flüchtigen Exanthem um eine akut rezidivierende Urticaria sensu stricto oder um ein urtikarielles, Vakzin-induziertes Exanthem handelt, kann letztlich nicht sicher differenziert werden.

Und auch ob die berichteten Laborveränderungen einer Vakzin-induzierten Immunreaktion oder einer subklinischen Virusinfektion zuzuordnen sind, ist aus unserer Sicht nicht vollständig zu klären.

Da die Patientin stets Wohlbefinden angab, sich die Haut- und Laborveränderungen spontan besserten und keine Sensibilisierung auf einen Inhaltsstoff nachgewiesen werden konnte, empfahlen wir eine weitere COVID-Impfung ohne Einschränkung.

## Fazit für die Praxis

- Unerwünschte Nebenwirkungen nach COVID-19-Impfungen zeigen eine große Variabilität; sie fallen in der Regel mild aus, können aber auch (selten) lebensbedrohlich sein.
- Unter den kutanen Nebenwirkungen zählen Lokalreaktionen an der Injektionsstelle, Urtikaria bzw. urtikarielle und morbilliforme Exantheme zu den häufigsten.
- Häufige Dermatosen (wie in unserem Fall eine Urtikaria bzw. ein urtikarielles Exanthem) können sich teils sehr ungewöhnlich präsentieren, somit auf die Patienten bedrohlich wirken und heben die Heterogenität der kutanen Nebenwirkungen nach COVID-19-Impfungen hervor.
